# COVID-19 Rehabilitation With Herbal Medicine and Cardiorespiratory Exercise: Protocol for a Clinical Study

**DOI:** 10.2196/25556

**Published:** 2021-05-26

**Authors:** Yang Gao, Linda L D Zhong, Binh Quach, Bruce Davies, Garrett I Ash, Zhi-Xiu Lin, Yibin Feng, Benson W M Lau, Peter D Wagner, Xian Yang, Yike Guo, Wei Jia, Zhaoxiang Bian, Julien S Baker

**Affiliations:** 1 Centre for Health and Exercise Science Research Hong Kong Baptist University Kowloon Hong Kong; 2 School of Chinese Medicine Hong Kong Baptist University Kowloon Hong Kong; 3 Hong Kong Chinese Medicine Clinical Study Centre Hong Kong Baptist University Kowloon Hong Kong; 4 Neurovascular Research Laboratory University of South Wales Pontypridd United Kingdom; 5 Veterans Affairs Connecticut Healthcare System West Haven, CT United States; 6 Center for Medical Informatics Yale University New Haven, CT United States; 7 School of Chinese Medicine Faculty of Medicine The Chinese University of Hong Kong New Territories Hong Kong; 8 School of Chinese Medicine The University of Hong Kong Hong Kong Hong Kong; 9 Department of Rehabilitation Sciences The Hong Kong Polytechnic University Kowloon Hong Kong; 10 Department of Medicine University of California San Diego La Jolla, CA United States; 11 Department of Computer Science Hong Kong Baptist University Kowloon Hong Kong; 12 Center for Translational Medicine and Shanghai Key Laboratory of Diabetes Mellitus Shanghai Jiao Tong University Affiliated Sixth People's Hospital Shanghai China

**Keywords:** COVID-19, rehabilitation, cardiorespiratory exercise, Chinese medicine

## Abstract

**Background:**

Recent studies have revealed that many discharged patients with COVID-19 experience ongoing symptoms months later. Rehabilitation interventions can help address the consequences of COVID-19, including medical, physical, cognitive, and psychological problems. To our knowledge, no studies have investigated the effects of rehabilitation following discharge from hospital for patients with COVID-19.

**Objective:**

The specific aims of this project are to investigate the effects of a 12-week exercise program on pulmonary fibrosis in patients recovering from COVID-19. A further aim will be to examine how Chinese herbal medicines as well as the gut microbiome and its metabolites regulate immune function and possibly autoimmune deficiency in the rehabilitation process.

**Methods:**

In this triple-blinded, randomized, parallel-group, controlled clinical trial, we will recruit adult patients with COVID-19 who have been discharged from hospital in Hong Kong and are experiencing impaired lung function and pulmonary function. A total of 172 eligible patients will be randomized into four equal groups: (1) cardiorespiratory exercise plus Chinese herbal medicines group, (2) cardiorespiratory exercise only group, (3) Chinese herbal medicines only group, and (4) waiting list group (in which participants will receive Chinese herbal medicines after 24 weeks). These treatments will be administered for 12 weeks, with a 12-week follow-up period. Primary outcomes include dyspnea, fatigue, lung function, pulmonary function, blood oxygen levels, immune function, blood coagulation, and related blood biochemistry. Measurements will be recorded prior to initiating the above treatments and repeated at the 13th and 25th weeks of the study. The primary analysis is aimed at comparing the outcomes between groups throughout the study period with an α level of .05 (two-tailed).

**Results:**

The trial has been approved by the university ethics committee following the Declaration of Helsinki (approval number: REC/19-20/0504) in 2020. The trial has been recruiting patients. The data collection will be completed in 24 months, from January 1, 2021, to December 31, 2022.

**Conclusions:**

Given that COVID-19 and its sequelae would persist in human populations, important findings from this study would provide valuable insights into the mechanisms and processes of COVID-19 rehabilitation.

**Trial Registration:**

ClinicalTrials.gov NCT04572360; https://clinicaltrials.gov/ct2/show/NCT04572360

**International Registered Report Identifier (IRRID):**

PRR1-10.2196/25556

## Introduction

In January 2020, the World Health Organization announced the outbreak of a novel coronavirus disease, called COVID-19. The COVID-19 pandemic has had devastating impacts globally, with 39,944,882 confirmed cases and 1,111,998 deaths recorded by October 20, 2020. Indeed, the number of cases of COVID-19 infection is continuously increasing with time, especially in the Americas, Europe, and Southeast Asia [1]. Although most people develop mild or uncomplicated forms of COVID-19, it has been estimated that approximately 14% of cases are associated with severe acute respiratory infection and may require hospitalization and oxygen support. Moreover, 5% of patients will require admission to an intensive care unit [2]. The COVID-19 pandemic has overwhelmed health systems worldwide. After discharge from hospital, some patients with COVID-19 continue to experience symptoms, which may last for months or even longer. These consequences include medical, physical, cognitive, and psychological problems. Although the long-term consequences are still unclear, recent studies have revealed that most patients (74%-87%) experience ongoing signs and symptoms 2-3 months after discharge from hospital, including fatigue, shortness of breath, impaired functions, and secondary mental problems [3,4]. Rehabilitation may contribute to patient recovery and is crucial in endowing patients with health improvements related to functional ability and quality of life.

For patients who have been unwell and sedentary for long periods, increased strength [5], aerobic ability [6], and exercise performance are vital to facilitate physiological [7], biochemical, psychological [8], and general recovery [9]. Considering the clinical conditions caused by prolonged immobilization and musculoskeletal deterioration, patients with COVID-19 need rehabilitation treatments following hospital discharge [10]. Exercise has direct effects on the cellular immune system; natural killer cells and T lymphocytes (T cells) become mobilized in the circulation through stress-induced shear stress and adrenergic signaling during exercise performance, providing protection and repair.

In addition to exercise intervention strategies, Chinese herbal medicines targeting gut microbiome dysbiosis have also been reported to improve immune function [11], facilitating patients’ recovery. The gut microbiome influences immune function and immune homeostasis both within the gut and systematically [12]. Chinese herbal medicines, the gut microbiome, and microbial metabolites regulate immune function and intestinal permeability involved in the pathological processes of virus-induced autoimmune diseases [11]. Restoring gut microbiome composition and its regulatory metabolites could be vital in boosting immune function and aiding recovery from COVID-19. Additionally, patients with COVID-19 experience loneliness, anxiety, depression, and decreased quality of life [13]. In summary, evidence suggests that exercise or Chinese medicines can help patients recover from virus-induced immune diseases and respiratory conditions such as pulmonary fibrosis. However, the potential beneficial individual and combined effects need further investigation.

In our proposed study, we will develop a new paradigm for the patient rehabilitation that is needed now and in the future. The specific aims of this project are (1) to investigate the effects of a 12-week programme involving cardiorespiratory exercise and Chinese herbal medicine, both singly and combined, on patients recovering from COVID-19 who exhibit pulmonary impairment and (2) to collect qualitative and quantitative data to examine the patients’ loneliness, anxiety, depression, quality of life, and mental health. A further aim will be to examine how Chinese herbal medicines as well as the gut microbiome and its metabolites regulate immune function, intestinal permeability, and possibly autoimmune deficiency in the pathological recovery and rehabilitation process. The specified study objectives and hypotheses are listed below:

1. Evaluate the effects of cardiorespiratory exercise and Chinese herbal medicine, both individually and in a combination, on rehabilitation on pulmonary function among patients with COVID-19 through four intervention groups, including a cardiorespiratory exercise plus Chinese herbal medicines group, a cardiorespiratory exercise group in isolation, a Chinese herbal medicines group, and a waiting list control group (primary outcome). We hypothesize that the combination of cardiorespiratory exercise and Chinese herbal medicines will more greatly facilitate the rehabilitation of patients with COVID-19.
2. Reveal whether and how the gut microbiome plays a role in the effects of cardiorespiratory exercise and Chinese herbal medicines, both individually and in combination, on the rehabilitation of impaired pulmonary function. We hypothesize that the combination of cardiorespiratory exercise and Chinese herbal medicines will better modulate the gut microbiome and the related metabolites and thus better mediate the effects.
3. Assess the effects of cardiorespiratory exercise and Chinese herbal medicines on immune functions, both individually and in combination, on the rehabilitation of other relevant symptoms and signs of COVID-19. We hypothesize that the combination of cardiorespiratory exercise and Chinese herbal medicines will better modulate the immune function and thus better mediate the effects.
4. Evaluate the effects of cardiorespiratory exercise and Chinese herbal medicines, both individually and in combination, on recovery from mental health-related outcomes (loneliness, stress, anxiety, depression) and on quality of life (QoL). We hypothesize that the combination of cardiorespiratory exercise and Chinese herbal medicines will better help postdischarge patients in recovery from mental health-related outcomes and improve their QoL.
5. Assess the lasting effects of cardiorespiratory exercise and Chinese herbal medicines after a 12-week follow-up period. We hypothesize that some effects (eg, dyspnea, mental health-related outcomes, QoL) will be sustainable, while others (outcomes sensitively responded to cardiorespiratory exercise, eg, physical fitness, lung function) will partially relapse after the intervention cessation.

## Methods

### Study Design

**Figure 1:**
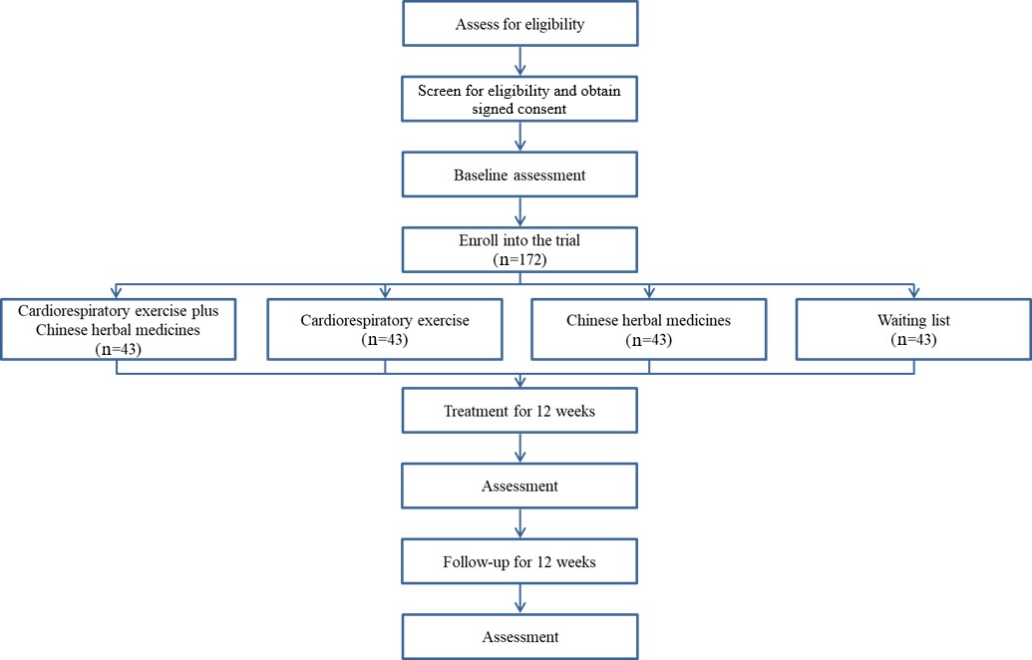
Flow diagram of the trial.

The proposed study will be a triple-blinded randomized controlled trial comprising four groups: (1) a cardiorespiratory exercise plus Chinese herbal medicines group, (2) a cardiorespiratory exercise group, (3) a Chinese herbal medicines group, and (4) a waiting list group. The target population will be postdischarge adult patients recovering from COVID-19 who were diagnosed with pulmonary impairments. Recruitment will take place in Hong Kong via our International Rehabilitation Network Center for Patients With COVID-19. The proposed rehabilitation period will last for 12 weeks, with a 12-week follow-up [14]. Primary outcomes will include the clinical symptoms of pulmonary fibrosis (dyspnea, fatigue, lung function, blood oxygen levels, immune function, blood coagulation, and related blood biochemistry). Blood biochemistry will be further analyzed using unique metabolomics techniques to identify potential synergies related to immune function response to the interventions [15]. We will investigate glucose metabolism and its association with the degree of infection and prognosis. We will also profile the gut microbial metabolome to establish associations between (virus-induced) dysbiosis and the outcomes of COVID-19 infection. Pulmonary function tests will be completed using clinical spirometry, as outlined by the American Thoracic Society. Blood gas levels, immune function, and coagulation levels of the patients will be measured pre- and post-intervention in venous blood samples [16]. The trial has been registered on ClinicalTrials.gov (NCT04572360). A flow diagram of the trial is shown in Figure 1.

### Eligibility Criteria

The target population will be adult patients with COVID-19 who have been discharged from the hospital and are still experiencing respiratory impairments. Patients will be recruited from Hong Kong. Inclusion criteria will include (1) age ≥18 years; (2) discharged from hospital for no less than 4 weeks; (3) percentage of predicted forced vital capacity (FVC) <90% or a percentage of predicted carbon monoxide diffusing capacity <90% [17]; (4) smartphone user; (5) able to communicate in Cantonese or Mandarin. Exclusion criteria will include (1) being pregnant or planning to become pregnant in the coming 6 months; (2) having acute exacerbations in the 12 weeks preceding recruitment; (3) having any contraindications for exercise (eg, physical disability, uncontrolled mental disorders, unstable heart disease, inability to perform muscle strength tests) [18]. The last two exclusion criteria will be evaluated by a physician.

### Randomization and Allocation Concealment

Participants will be randomly and equally assigned to the four groups using block randomization [19]. The random allocation sequence will be computer generated by a blinded statistician outside of the research team, who will use randomly mixed block sizes of 4 and 8 and conceal the randomization procedure from the research team. The results of the allocation will be randomly coded with an identification number according to the random number table. An investigator will dispense the identification numbers according to the order of the patient’s recruitment and will not be allowed to arbitrarily alter the numbering.

### Blinding

One copy of the blind codes will be held by the project-responsible unit. The investigator operating the allocation of patients will not be involved in the intervention delivery, outcome measurements, or data analysis. The results of the allocation (being in the intervention or control groups) will be concealed from patients due to the waiting list control design, where patients in the control group will also receive a type of intervention and therefore will not know they are control participants. In addition, investigators assessing effects and those analyzing the data will also be blinded from the allocation.

### Interventions

#### Cardiorespiratory Exercise Intervention

This intervention will be a 12-week progressive and individualized exercise program with a frequency of 3 sessions per week and a session duration of 60 minutes (total: 36 exercise sessions). The intervention is designed to alleviate symptoms of dyspnea, improve functional capacity, and relieve mental health problems in the short term. A further aim is to preserve physical function while improving health-related QoL in the long term. As recommended by the Official American Thoracic Society/European Respiratory Society Statement [20], 12 education sessions (30 minutes per session) will be included in and delivered together with the first exercise session in each week to motivate and empower the patients to form regular exercise habits and improve health [21,22]. A steering group will be formed to design the exercise intervention, develop relevant documentation, and monitor the implementation. Group members will include exercise experts, public health specialists, occupational therapists, doctors, and other stakeholders. Textbox 1 and Table 1 provide a summary of the intervention.

This program will be delivered using a home-based tele-exercise approach to reduce the potential to infect other individuals [24], reduce health care burden and costs for both health care providers and patients, and improve participation and retention of rehabilitation [25]. Some evidence, although not for COVID-19, has suggested that home-based pulmonary rehabilitation with minimal exercise equipment is effective and may be a viable alternative to equipment-intensive clinic-based programs [18,26]. Other studies concluded that the web-based tele-exercise approaches were as effective as conventional face-to-face interventions [27] and could enhance the reach and engagement of the rehabilitation process [21,25]. We will build and develop a web-based platform in our International Rehabilitation Network Center for Patients With COVID-19 to deliver the intervention.

**Description of the cardiorespiratory exercise intervention (educational session).**

**Educational session**

- Each session will last 30 minutes and will be held before the first exercise session of each week, for a total of 12 sessions.
- The sessions will be developed mainly based on social psychological theories of intentional behavior and motivation, and aimed to improve motivation, self-regulation, and habit-formation relating to exercise [20,22,23].
- Our team members published a similar theory-based behavior change intervention in 2020 [22]. In brief, in each session, the patients will be asked about their exercise and other types of physical activity (PA), related theoretical determinants (eg, self-efficacy), and other related factors (eg, social and environmental factors). They will then receive personally tailored feedback incorporating effective behavioral change techniques (eg, goal setting) to promote motivation, solve problems, make action plans, prevent relapse, enhance self-regulation, and form healthy habits. A recap of the previous session will be added starting in the second session. Pictures, graphs, and videos will also be used where needed.

**Table 1:** Description of the cardiorespiratory exercise intervention (exercise session).

Content	Description
Exercise session	3 sessions per week for a total of 36 sessions; session duration will gradually increase from 40 to 60 minutes. Initial face-to-face briefing will be given to enhance compliance and ensure that the patients can exercise properly. Video feedback will be provided during each session.
Warm-up	Warm-up will last 5-8 minutes, including both static stretching and dynamic stretching.
Aerobic training	There will be no requirement of intensity in the initial sessions (1-2 weeks). Exercise intensity will start from Rating 3 of the Borg Category-Ratio 0-10 Scale (Borg Dyspnea Scale) and progressively increase to Rating 6. The training mode will be interval training in the first 4-6 weeks, followed by continuous training. The duration will gradually increase from 10 to 30 minutes. Effective exercises will be selected after a thorough review of the existing literature, such as walking, high knees, butt kicks, and stepping.
Resistance training	The intensity will be one that evokes local muscular exhaustion in 6 to 12 repetitions (ie, a 6-12 repetition maximum) for major muscle groups; workload will increase when an individual can perform the current workload for 1 or 2 more repetitions than the required number in two consecutive exercise sessions [23]; there will be no requirement of intensity in the initial sessions (1-2 weeks). The volume will be 2-4 sets, 8-12 repetitions, with 1-minute rest intervals (approximately 10 minutes). A resistance band will be used to strengthen the muscles of either upper or lower extremities alternatively in each session. Effective exercises will be selected after a thorough review of existing literature. Exercise examples include band bicep curl (arms), band lateral pull (arms), lateral band walk (legs), and seated banded leg extensions (legs).
Cool-down	Cool-down will last 5-8 minutes, including static stretching and dynamic stretching.
Inspiratory muscle training	The device used will be a POWERbreathe Classic (IMT Technologies Ltd). The volume and intensity will be 30 breaths at 50% of maximal inspiratory pressure (approximately 5-8 minutes). The increment of intensity will be 5% load increase each week. Additional Inspiratory muscle training sessions will be performed without supervision according to a suggested frequency of twice daily. Maximal inspiratory pressure will be measured with a portable hand-held mouth respiratory pressure meter (MicroRPM, CareFusion Micro Medical).

#### Structure of the Exercise Intervention

The intervention will include the following components: (1) a set of home-based tele-exercise sessions during which remote monitoring of vital signs will be required; (2) an individualized action plan to perform various daily physical activities; (3) educational sessions on self-management and habit formation; (4) access to a call center; (5) counselling sessions to enhance motivation to regularly engage in daily physical activities. The counseling intervention will be facilitated via a remote patient monitoring system. Patients will be equipped with an activity monitor (Mi Band 5, Xiaomi Corporation) that connects to a customized smartphone or tablet with a global system for mobile communication (GSM) connection that will receive, display, and send the data via the internet to a secure web-based server at the university. Patients will receive real-time feedback on the smartphone or tablet about their physical activity levels and their daily adjusted physical activity goals. A teleconference between the patient and the counselor will also be possible via the smartphone or tablet (using Zoom) in order to continually enhance adherence and goal achievement. Patients’ daily physical activities will be acquired by the Mi Band 5 via Bluetooth technology to the smartphone or tablet; from the smartphone or tablet, data will be sent to the central server. Following the completion of the intervention, all patients will receive detailed individual feedback in relation to the outcome measures obtained via a meeting with the research team. This will comprise a question and answer session with relevant experts in their field of expertise. Patients will also receive a detailed personalized report with recommendations and information relating to the outcomes of the intervention.

The content of the exercise intervention will be developed with reference to the Official American Thoracic Society/European Respiratory Society Statement published in 2013 [20]. Each exercise session (40-60 minutes) will include a warm-up, aerobic training, resistance training, a cool-down, and inspiratory muscle training (IMT) [20,28,29]. Aerobic exercise will be arranged before resistance training, as recent evidence has shown that this order produces greater benefits than performing resistance training first [30]. Additional IMT sessions will be performed without supervision according to a suggested frequency of twice daily [20]. Before the first exercise session of each week, an additional educational session (30 minutes) will be delivered to improve motivation, self-regulation, and habit formation relating to exercise [20]. The contents of the educational sessions will be developed mainly based on social psychological theories of intentional behavior and motivation and effective behavioral change techniques (Textbox 1) [20,22]. All patients will be briefed face-to-face initially to ensure that they can undertake the intervention properly and to enhance their compliance with the intervention. In addition, they will be videotaped during the exercise sessions, and real-time feedback will be given via the internet.

Aerobic exercise intensity will be determined using the Borg Category-Ratio 0-10 Scale (Borg CR10 Scale) modified for dyspnea, as recommended by the American College of Sports Medicine guidelines for patients with pulmonary diseases [23]. The exercise intensity will start from Rating 3 (equivalent to 53% peak work rate, ie, moderate intensity) in the first 2-4 weeks and progressively increase to Rating 6 (equivalent to 80% peak work rate, ie, vigorous intensity). Exercise intensity and session duration will be adjusted according to each patient’s response and tolerance. Lighter intensities than Rating 3 may be adopted when there is a need (eg, for those with severe conditions). Patients will be given specific and standardized instructions on the ratings in advance. A pulse oximeter (Beurer PO 40, Beurer GmbH) will be used to monitor oxygen saturation in the initial exercise sessions to avoid exercise-induced oxyhemoglobin desaturation. In terms of intensity of resistance training, a practical approach to determine the appropriate intensity will be adopted, in which workloads will be set at levels that evoke local muscular exhaustion in 6 to 12 repetitions, as the one repetition maximum approach may overestimate or underestimate the optimal resistance for individuals [20,31]. Progressive target goals and detailed training plans will be set up together with each patient based on their physiological measurement results [21].

The exercise intervention will be delivered and supervised by experienced instructors. We will recruit persons who have been educated in either exercise science or rehabilitation. Each exercise session will be facilitated by two instructors dedicated to a group of 5-7 patients through the web-based platform in our International Rehabilitation Network Center for Patients With COVID-19. They will also be responsible for monitoring developments and training during the intervention.

#### Chinese Herbal Medicines

The Chinese herbal formula of Modified Bai He Gu Jin Tang (including Rehmanniae Radix 10 g, Rehmanniae Redix Praeparata 10 g, Ophiopogonis Radix 10 g, Lilii Bulbus 15 g, Paeoniae Radix Alba 10 g, Angelicae Sinensis Radix 10 g, Fritillariae Thunbergii Bulbus 5 g, Glycyrrhizae Radix et Rhizoma 10 g, Platycodonis Radix 15 g, and Salviae Miltiorrhizae Radix et Rhizoma 15 g) will be prescribed in granules. The rationale for the formula selection is as follows: (1) based on the syndrome differentiation, pulmonary fibrosis post–COVID-19 appears as qi-yin deficiency of the lung [32]; (2) a previous study has shown that many herbs using this formula and Salviae Miltiorrhizae Radix et Rhizoma have antifibrosis effects [33]; and (3) Bai He Gu Jin Tang has the effects of nourishing lung qi and ying deficiency [34]. Therefore, the formulation of Bai He Gu Jin Tang was modified to match the therapeutic purpose. A dose of 10 g per day (5 g, twice per day) will be ingested. Patients will dissolve a sachet of granules (5.0 g) in 200 ml of hot water twice per day after breakfast and dinner, 7 days per week, for 3 months. The entire manufacturing process will be in strict compliance with the standards of good manufacturing practice.

#### Waiting List Control

The waiting list control sign will be adopted to conceal the allocation results from the patients as well as to reduce selection and confounding bias and increase the participants’ adherence to the study. Patients in the waiting list control group will receive no treatment in the study period (including a 12-week intervention period and a 12-week follow-up period). However, they will receive Chinese herbal medicines after the completion of the study (ie, after the third wave of measurements in the 25th week).

### Outcomes

Table 2 lists a description of the outcomes and other measures, which are not exhaustive. All measurements will be recorded at baseline and repeated at the 13th and 25th weeks of the study. Patients will be assessed for risk prior to data collection. Further information from patients using appropriate data collection methodologies, including clinical symptoms, treatment history, comorbidities, physical activity, nutrition, and other potential variables, will be obtained and adjusted for during data analysis.

**Table 2:** Description of the outcome measures.

Measure		Outcomes
Cardiorespiratory fitness		Six-minute walk test Performance time test
Dyspnea		Borg Category-Ratio 0-10 Scale (Borg Dyspnea Scale)
Body composition		BMI Waist circumference Stature Body mass Segmental muscle mass Anatomical circumference
Lung function test		Forced vital capacity Forced expiratory volume 1 Forced expiratory volume 1/forced vital capacity Fractional exhaled nitric oxide DLCO^a^
Cardiopulmonary exercise test/blood gas		VE^b^ VCO_2_^c^ VO_2_^d^/PCO_2_^e^ PO_2_^f^
**Blood biochemistry tests**		
	Metabolic function	Insulin
	Immune function	Coagulation Lymphocytes CD3 CD4 CD8 CD19 CD16+CD56 T cells
	Neurological function	BDNF^g^ Corticosterone
	Cytokine profiles	Interleukin 1 Interleukin 6 Tumor necrosis factor alpha
Gut microbiome		Fecal metagenomics analysis
Glucose, fructose, and related metabolites		UPLC-QTOF-MS^h^ analysis
Metabolomics-related measurement of depression		Metabolomics analysis of selected neurotransmitters as potential markers of depression
Quality of life and other mental health–related measures		Self-reported scales

^a^DLCO: diffusing capacity of the lungs for carbon monoxide.

^b^VE: ventilation.

^c^VCO_2_: carbon dioxide output.

^d^VO_2_: oxygen uptake.

^e^PCO_2_: partial pressure of carbon dioxide.

^f^PO_2_: partial pressure of oxygen.

^g^BDNF: brain-derived neurotrophic factor.

^h^UPLC-QTOF-MS: ultra-performance liquid chromatography coupled with quadrupole/time-of-flight mass spectrometry,

### Outcome Measures

#### Cardiorespiratory Fitness

Functional exercise capacity that reflects daily physical activities will be assessed using the six-minute walk test (6MWT) to assess the outcome of the intervention. This test is recommended by the American Thoracic Society to evaluate the global and integrated responses of all the systems involved during exercise, including the cardiopulmonary systems, systemic and peripheral circulation, blood, neuromuscular units, and muscle metabolism. Heart rate and blood pressure will be measured before and after exercise. The test has high test-retest reliability (intraclass correlation coefficient=0.88-0.91) [35].

#### Dyspnea

Dyspnea is a common exercise-induced symptom of disease and usually occurs in patients with pathophysiology that results in inefficient gas exchange and ventilator impairments. The Borg CR10 Scale (Borg Dyspnea Scale) will be used to rate the dyspnea and overall fatigue levels of the patients at the beginning and end of the 6MWT. The scale will also be used during the cardiopulmonary exercise test (CPET).

#### Body Composition

Segmental muscle mass and BMI (kg/m^2^) will be used to assess the body composition of the patients. A bioimpedance analysis approach (InBody 770 analyzer, InBody Co, Ltd) will be used to assess the patients’ segmental muscle mass. A stadiometer (Seca 284, Seca Corporation) will be used to measure stature and body mass. Anatomical circumference will be measured using a distensible measuring tape.

#### Lung Function

The FVC test, forced expiratory volume (FEV_1_), and FEV_1_/FVC ratio will be assessed to indicate the functional severity and capacity of the patients’ lungs (Vmax Encore V229, VIASYS Respiratory Care Inc). Fractional exhaled nitric oxide (FeNO) will be used to assess inflammatory response to exercise and medicinal intervention (NObreath, Bedfont Scientific Ltd, England).

#### Cardiopulmonary Exercise

The CPET will be performed using the Vmax Encore V229. The CPET provides information concerning the level of exercise that the patient can perform without undue stress. The test results will guide the research team regarding the prescription of exercise for physical rehabilitation methodologies. It also provides quantitative evidence of the benefits of a rehabilitation program as well as information on the mechanisms involved. Improvement in exercise tolerance cannot be objectively assessed without CPET [36]. An incremental ergometry exercise test (Excalibur ergometer, Lode Ergometry), will be used to assess the responses of the cellular, cardiovascular, and ventilatory systems under precise conditions of metabolic stress. Breath by breath measurements of minute ventilation (VE), carbon dioxide output (VCO_2_), O_2_ uptake (VO_2_), VE/VCO_2_, VE/VO_2_, deviation in the responses from the pulmonary to cellular respiration and arterial blood gas, partial pressure of carbon dioxide (PCO_2_), and oxygen saturation as measured by pulse oximetry (SpO_2_, Sentec AG), will be monitored continuously during the exercise test. Depending on the physical condition of the patients, modification of the protocols to smaller work rate increments may be warranted. Intensities and durations will be specific to each individual to facilitate measurement and data capture. Before each test, the O_2_ and CO_2_ analyzers of the CPET system will be calibrated using standard gases (Gas 1: O_2_: 25.89%, CO_2_: 0%; Gas 2: O_2_: 16.44% CO_2_: 3.89%), and the flow volume integration system will be was calibrated by applying a standard volume (3 L) of gas at various flow rates. Canopy studies within the Vmax software suite will be used to validate the accuracy of the gas-exchange measurement instruments that measure VO_2_ and VCO_2_ using the methanol burning technique. The ratio of VCO_2_ to VO_2_ in the combustion of methanol yields a respiratory quotient (RQ) of 0.667. We will use a 3% difference as the accuracy threshold for measured RQ (RQ range of 0.647-0.687).

#### Quality Assurance, Calibration, Accuracy, and Precision for the Laboratory-Based Assessment Measures

All the devices used for measurement will be calibrated according to the manufacturer's specifications. The protocols of the measurements and sources of variability will be controlled by following the standards outlined in the American Thoracic Society calibration guidelines for spirometers and gasometers. Anatomical circumference measurements will be adopted from the anthropometry procedures manual provided by the US Centers for Disease Control and Prevention [37]. Repeated tests will be performed at the same time of day in a controlled laboratory environment of constant humidity, temperature, and pressure. Patients will arrive at the laboratory at designated times for all physiological and clinical assessments. All patients will be transported to the laboratory for testing and returned home by coach. A recovery period will be observed postassessment to ensure that the participants have no negative responses to the measurements. Once the recovery period is complete, all participants will be returned home.

#### Blood Biochemistry

Venous blood will be drawn at 3 time points, namely at pretreatment, at posttreatment, and after the 12-week follow-up. After coagulation at room temperature for 30 minutes, the samples will be centrifuged at 3000 rpm for 20 minutes. Serum as the supernatant will be extracted and stored at –80 ˚C until assay. Serum levels of insulin will be assessed by a commercially available enzyme-linked immunosorbent assay (ELISA) kit (Merck & Co). Serum will be isolated by centrifugation (1000 rpm for 30 minutes, 4 °C) and stored at –80 °C until analysis. The prothrombin time test will be used to measure blood coagulation. Venous blood will be collected by venepuncture in a tube with sodium citrate. Plasma will be isolated after centrifugation at 1000 rpm for 30 minutes at 4 °C. Thromboplastin will be added to the plasma and maintained at 37 °C for 2 minutes. Calcium chloride will be added to the mixture, and the plasma will be allowed to coagulate. The time needed for the coagulation will be recorded as the prothrombin time.

Flow cytometry for counts of main lymphocyte subpopulations (CD3, CD4, CD8, CD19, and CD16+CD56 T cells) will be performed to test immune function. Venous blood will be stored in tubes with EDTA (anticoagulant). 100 µL of blood will be stained within 24 hours of sampling by the BD Multitest 6-color TBNK reagent (BD Biosciences). The lymphocytes with the antigens CD3, CD4, CD8, CD19, and CD16+CD56, which are the main populations of lymphocytes, will be labelled by a fluorochrome-conjugated antibody in the reagent. The whole blood will be incubated in the reagent at room temperature for 30 minutes, and red blood cells will be lysed with ammonium chloride solution. Cell sorting and analysis will be performed with a BD Accuri C6 flow cytometer (BD Biosciences). Each subpopulation of lymphocytes will be expressed in absolute value and percentage of total lymphocytes.

As suggested by Hacimusalar and Eşel [38], the following markers related to emotional disturbances will be studied.

##### Brain-Derived Neurotrophic Factor

Serum concentration of brain-derived neurotrophic factor (BDNF) will be measured with a commercially available ELISA kit (Millipore) according to the manufacturer’s instructions.

##### Corticosterone

Serum concentration of corticosterone will be assayed by ELISA kit (Enzo Life Sciences, Inc) according to the manufacturer’s instructions.

##### Cytokine Profile

The serum concentrations of interleukin-1, interleukin-6, and tumor necrosis factor alpha will be assayed by multiplex cytokine analysis. A multiplex cytokine kit (20-plex, Bio-Rad Laboratories) will be used according to the manufacturer’s instructions. The result will be generated by the Bio-Plex 200 suspension array system (Bio-Rad Laboratories) that is available in the University Life Science facility of Hong Kong Polytechnic University.

#### Gut Microbiome Test

All patients will be requested to self-sample their first morning feces by following detailed printed instructions. Collected stool samples will be immediately frozen in a home freezer (–20 ℃), then transported to our facilities in a provided freezer pack and stored at –80 ℃ for long-term use. Total DNA of 200 mg fecal samples will be extracted and purified as described [39]. The DNA concentrations and size distributions will be estimated using a NanoDrop instrument (Thermo Scientific) and agarose gel electrophoresis, respectively. The DNA one-paired-end library will be prepared using a TruSeq DNA HT Sample Prep Kit (Illumina Inc), and whole-genome shotgun sequencing of the samples will be carried out using the HiSeq 2000 platform (Illumina Inc). Low-quality reads with N bases, adapter contamination, or human DNA contamination will be filtered from the raw data, and the remaining high-quality sequences will be mapped with the published gene catalog of reference genes in the human gut microbiome [40]. Taxonomic assignment of the predicted genes and

Kyoto Encyclopedia of Genes and Genomes (KEGG) analysis will be performed as described previously [41]. The relative abundances of phyla, genera, species, and KEGG orthologs will be calculated from the relative abundances of the respective genes. For data analysis, the quantitatively measured metabolites for each participant will be merged into the participants’ clinical information through their individual sample IDs.

#### Mass Spectral Analysis of Glucose, Fructose, and Related Metabolites

Human serum or plasma samples will be thawed on an ice bath to minimize sample degradation. Metabolites will be extracted by adding 0.5 mL of 50% methanol (–20 °C) followed by homogenization for 3 minutes using a Bullet Blender Tissue Homogenizer (Next Advance, Inc) and centrifugation at 13,500 g for 10 minutes at 4 °C. The resulting 50 μL of supernatant will be transferred to a 1.5 mL tube and mixed with 10 μL of 200 mM 3-nitrophenylhydrazine (3-NPH) solution and 10 μL of mixed 96 mM ethylene dichloride/pyridine methanolic solution. Derivatization will be conducted by incubation at 30 °C for 1 hour before evaporation to dryness under nitrogen. 400 μL of 50% aqueous methanol will be used to resuspend the samples. The supernatants will be used for ultra-performance liquid chromatography coupled with quadrupole/time-of-flight mass spectrometry (UPLC-QTOF-MS) analysis according to previous reports with minor modifications [42,43].

#### Metabolomics-Related Measurement of Depression

##### Metabolomics Analysis

Ultra-performance liquid chromatography triple quadrupole mass spectrometry (UPLC-TQ-MS) (Xevo TQ-S system, Waters Corp) will be used to quantitatively measure the metabolites (neurotransmitters) selected as potential markers of depression. Briefly, an aliquot of 40 μl of urine or plasma will be spiked with 10 μL of internal standard (L-4-chlorophenylalanine in water, 30 μg/mL) and extracted with 200 μL of acetonitrile and methanol (9:1, v/v). The mixture will be vortexed and centrifuged. After centrifugation, the supernatant will be transferred to the sampling vials and subject to UPLC-TQ-MS analysis. The raw data generated will be processed using the TargetLynx Applications Manager Version 4.1 (Waters Corp) for targeted metabolite annotation and to obtain the calibration equations and the concentration of each metabolite in the samples.

##### Quality Control

Reproducible and valid results are critical for biomarker development. To achieve this, three types of quality control (QC) samples will be used in our metabolomics analysis: internal standards, test mixtures, and pooled biological samples. In addition to these QC samples, conditioning and solvent blank samples are required to obtain optimal instrument performance. Test mixtures comprise a group of commercially available standards with a mass range across the system mass range used for the study samples. These samples are analyzed at the beginning and end of each batch run to ensure that the instruments are performing within analytical specifications, such as retention time stability, peak resolution, peak signal intensity, and mass accuracy. Internal standards will be added to the test samples to monitor analytical variations during the entire sample preparation and analysis processes. The quality assurance criteria used to monitor the internal standards include (1) coefficient of variation (CV) ≤15% within 100 injections; (2) CV ≤20% within 300 injections. The CV is defined as the ratio of the standard deviation to the mean peak signal intensity. A pooled plasma sample containing aliquots from representative participants will be used as a study QC sample for the correction of interbatch analysis. The QC samples for this project will be prepared with the test samples and injected at regular intervals, every 12 testing samples, throughout each analytical run. The purpose of using the pooled QC samples is to provide a set of data that can be used to assess overall reproducibility and to correct for potential analytical variations. To minimize the batch-to-batch and day-to-day variations, we will attempt to arrange one case and one control sample adjacent to each other in each batch and on each day of testing. (3) Data assembly: for data analysis, the quantitatively measured metabolites for each participant will be merged with the participant’s clinical information through their individual sample IDs.

#### QoL and Other Mental Health-Related Measures (Self-Reported)

QoL will be measured using the Personal Well-being Index-Chinese Version. It is a subjective QoL measure that has been translated and validated. Anxiety will be measured using the Chinese version of the Depression Anxiety Stress Scale-21, which has been proven to have internal consistency [44,45]. The scale will discriminate between the negative emotional syndromes of depression, anxiety, and stress in Chinese populations. Only the subscales of anxiety and stress will be used. Loneliness will be measured using the Revised UCLA Loneliness Scale) [46,47]. This scale consists of 20 items and has been widely used to assess loneliness in research. General mental health will be measured using the General Health Questionnaire, which is commonly used to screen minor psychiatric symptoms [48].

### Safety Observations

During the trial, each participant will receive safety monitoring. All adverse events will be forwarded to the ethics committee. They will review all documented harms during the trial and adjudicate them with regard to causality. The observations are listed below:

1. Vital signs: Body temperature, heart rate, blood pressure, and respiration (once at each follow-up visit)
2. Blood routine, urine routine, stool routine plus occult blood (once before and after treatment, respectively; twice in total)
3. Hepatic function (alanine aminotransferase, aspartate aminotransferase, total bilirubin, alkaline phosphatase, gamma-glutamyl transferase), renal function (serum creatinine, blood urea nitrogen) (once before and after treatment, respectively; twice in total)
4. Electrocardiogram (once before and after treatment, respectively; twice in total)
5. Severity and incidence of adverse events (recorded in detail at any time)

The schedule for the enrollment, interventions, and assessment is summarized in Table 3.

**Table 4:** Schedule of enrollment, interventions, and assessments in the trial.

Time point		Study period (week)				
		Enrollment		Allocation	Post-allocation	Follow-up
		Washout (–3 to –1)	Run-in (–1)	0	12	12
**Enrollment**						
	Eligibility screen	✓				
	Informed consent	✓				
	Randomization			✓		
	Allocation			✓		
**Intervention**						
	Cardiorespiratory exercise plus Chinese herbal medicines				✓	
	Cardiorespiratory exercise				✓	
	Chinese herbal medicines				✓	
	Waiting list					
**Assessment**						
	Cardiorespiratory fitness				✓	✓
	Dyspnea				✓	✓
	Body composition				✓	✓
	Lung function test				✓	✓
	Cardiopulmonary exercise test				✓	✓
	Blood biochemistry tests				✓	✓
	Gut microbiome				✓	✓
	Glucose, fructose, and related metabolites				✓	✓
	Metabolomics-related measurement of depression				✓	✓
	Quality of life and other mental health-related measures				✓	✓
	Safety	✓	✓	✓	✓	✓

### Infectious Control

Prior to and following the testing procedures, all equipment will be subjected to rigorous disinfection methods for clinical populations as outlined by the Guidelines on Infection Control Practice in the Clinical Setting of the Department of Health [49]. Further to this, individuals involved in testing and data collection will be required to wear the necessary protective clothing and observe personal hygiene regulations as further outlined in the document [49].

### Data Management and Storage

The original medical records will be kept intact by the site investigators as the original documents of the clinical trial. All researchers will receive training regarding data management. Web-based application and user sessions will be encrypted between the server and client browser through the use of industry standard secure sockets layer (SSL) certificates. The data will be entered into the electronic case report form and the database will be established before recruitment. The investigators will be responsible for verifying the accuracy of the data. Data locking will be completed by the data management team, and the researchers will not be able to modify the data. All research documents, including paper and electronic documents, will be retained for at least 5 years after publication. All individual participant data collected during the trial will be deidentified and made available to anyone who wishes to access the data immediately following publication.

A Data and Safety Monitoring Board (DSMB) has been set up to review the protocol and the research data. The DSMB will meet regularly to review the protocol according to ethical and safety standards, monitor the safety of the trial, and monitor the authenticity and completeness of the data based on the study design. The DSMB will review the progress of the trial, determine adverse events, and have the authority to decide whether the study needs to end early.

### Data Protection

All data protection and patient anonymity considerations will apply throughout the experimental and postexperimental period. All patients prior to participation will be required to complete an informed consent form, clearly outlining experimental procedures and information relating to the study. All data will be anonymized with the unique experimental IDs provided to the patients. Data collected will be stored in a secure location under lock and key. Computer-generated information will be given specific passwords, with access granted to the principal investigator. Patients will be informed that they can withdraw from the study at any time. Prior to data collection, ethical approval will be obtained from the ethics committee. All data collection procedures, confidentiality issues, and patient integrity will be subject to methodological rigor as outlined by the data protection act.

### Sample Size Calculation

Aiming to achieve a large effect size of 0.8, based on a meta-analysis of effectiveness of exercise interventions on dyspnea and exercise capacity in patients with pulmonary fibrosis diseases [28], using a significance level of 0.05, power level of 90%, and 20% drop-out rate, a sample size of 172 patients (43 per group) was preliminarily estimated for the study. The meta-analysis result was chosen because there is no similar rehabilitation program available that combines exercise and Chinese herbal medicine. Furthermore, the outcome variables in that meta-analysis are similar to our primary outcomes. Given that the effects of Chinese herbal medicine were not considered, the final sample size will be re-estimated using preliminary outcomes from the pilot study.

### Statistical Analysis

A generalized linear mixed model will be used to compare between-group differences in changes of outcome variables at the 12- and 24-week periods of the study, where group and time are the two factors of interest. Mediating effects of the gut microbiome and immune function tests will be determined by fitting linear regression models for pulmonary and other outcomes, respectively. A significance level of 0.05 will be adopted (two-tailed test). Intention-to-treat procedures will be applied to the data sets obtained. Missing data will be imputed using multiple imputations with chained equations, except for those from dropouts, which will be imputed with the baseline data. Data will be scrutinized for normal distribution prior to data analysis. Sensitive analyses, such as per-protocol analysis, as-treated analysis, and analysis for complete cases, will only be performed to assess the robustness of the data analyses.

### Data Sharing

All individual participant data collected during the trial will be deidentified and will be available to anyone who wishes to access the data immediately following publication.

## Results

The trial was approved by the university ethics committee following the Declaration of Helsinki (REC/19-20/0504) in 2020. The trial has been recruiting patients. The data collection will be completed in 24 months, from January 01, 2021, to December 31, 2022.

## Discussion

As revealed in recent studies, long-term (12 weeks or more) interval exercise can significantly improve ventilatory, central hemodynamic, and peripheral muscle capacities [50]. During the COVID-19 pandemic, home-based tele-coaching techniques, such as a smartphone and step counter, could provide a comfortable and safe environment for rehabilitation that is as effective as that of hospital-based rehabilitation [51-53].

Because of the focus on rehabilitation, using a combination of exercise and Chinese medicine, and exploring the role of the gut microbiome in a mechanistic manner, the proposed study would be the first of its kind for this type of experimental intervention. Given that COVID-19 could persist in human populations, the important findings from this study would provide valuable insights into the mechanisms and processes that are active during rehabilitation.

The proposed multiple components of the rehabilitation program will benefit patients post–COVID-19 who use the various treatment modalities in the process. The findings and implications of this study will have long-lasting positive health benefits for patients with COVID-19 and will help treat related comorbidities. The findings from the study will also provide economic comparative data relating to the development of a cost-effective model for postevent rehabilitation of patients with COVID-19.

## Abbreviations

3-NPH: 3-nitrophenylhydrazine
6MWT: six-minute walk test
BDNF: brain-derived neurotrophic factor
Borg CR10 Scale: Borg Category-Ratio 0-10 Scale
CPET: cardiopulmonary exercise test
CV: coefficient of variation
DSMB: Data and Safety Monitoring Board
ELISA: enzyme-linked immunosorbent assay
FeNO: fractional exhaled nitric oxide
FEV1: forced expiratory volume
FVC: forced vital capacity
GSM: global system for mobile communication
IMT: inspiratory muscle training
KEGG: Kyoto Encyclopedia of Genes and Genomes
PCO_2_: partial pressure of carbon dioxide
QC: quality control
QoL: quality of life
RQ: respiratory quotient
SpO_2_: oxygen saturation as measured by pulse oximetry
SSL: secure sockets layer
UPLC-QTOF-MS: ultra-performance liquid chromatography coupled with quadrupole/time-of-flight mass spectrometry
UPLC-TQ-MS: ultra-performance liquid chromatography triple quadrupole mass spectrometry
VCO_2_: carbon dioxide output
VE: ventilation
VO_2_: O_2_ uptake
